# Knowledge, attitudes, and practices with respect to epilepsy among primary and secondary school teachers in the city of Niamey, Niger

**DOI:** 10.1002/brb3.1539

**Published:** 2020-01-28

**Authors:** Hamid Assadeck, Moussa Toudou Daouda, Mahadi Moussa Konate, Zakaria Mamadou, Dijbo Douma Maiga, Samuila Sanoussi

**Affiliations:** ^1^ Department of Neurology National Hospital of Niamey Niamey Niger; ^2^ Department of Medicine and Medical Specialties Faculty of Medicine and Pharmacy Abdou Moumouni University Niamey Niger; ^3^ Department of Neurology General Referral Hospital of Niamey Niamey Niger; ^4^ Department of Psychiatry National Hospital of Niamey Niamey Niger; ^5^ Department of Neurosurgery National Hospital of Niamey Niamey Niger; ^6^ Department of Surgery and Surgical Specialties Faculty of Medicine and Pharmacy Abdou Moumouni University Niamey Niger

**Keywords:** attitudes, epilepsy, knowledge, Niamey, Niger, practices, school teachers

## Abstract

**Objective:**

We aimed firstly to evaluate knowledge, attitudes, and practices about epilepsy among primary and secondary school teachers in the city of Niamey and secondly to formulate targeted sensitization messages for these teachers.

**Materials and methods:**

A descriptive cross‐sectional survey was carried out among primary and secondary school teachers in the city of Niamey, capital of Niger, using a self‐administered questionnaire including questions related to knowledge, attitudes, and practices about epilepsy.

**Results:**

One hundred and forty‐five (145) teachers aged 27–69 (mean age: 39.57 ± 8.304 years) had answered the questionnaire (52 men and 93 women). In 62.1% of cases, respondents had a bachelor degree in education signifying a high level of education. Sixty‐one respondents (42.1%) considered epilepsy as a brain disease, whereas it was considered as a psychiatric illness by 15.9% of respondents and as an impurity by 38.9%. Sixteen respondents (11%) considered it as a hereditary disease. Epilepsy was considered as a contagious disease by 46.2% of teachers, and the main transmission routes reported were contact with places of crisis (26.2%), physical contact with an epileptic person (16.6%), and contact with saliva (6.9%). It was considered as an incurable disease by 6.9% of teachers. Of the 115 respondents (79.3%) who considered epilepsy as a treatable condition, 46 of them believe that epilepsy is treated by traditional medicine. When someone has an epileptic seizure, 28.3% of respondents think that physical contact with him should be avoided and that the places of the crisis should be avoided.

**Conclusion:**

Considering that epilepsy is common in school‐age, the study results suggest the need to train teachers with respect to epilepsy to change misconceptions about epilepsy and to promote positive attitudes toward epileptic people to avoid the rejection of these people by the society responsible for problems of social integration.

## Graphical abstract

The study evaluates knowledge, attitudes, and practices about epilepsy among school teachers of primary and secondary schools in the city of Niamey, capital of Niger. The study results revealed a low level of general knowledge about epilepsy among respondents, of whom only 42.1% think that epilepsy is a brain disease. 15.9% and 46.2% of the respondents considered epilepsy to be a psychiatric illness and a contagious disease, respectively. 6.9% of the respondents considered epilepsy to be an incurable disease.

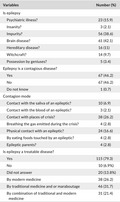

## 1 INTRODUCTION

One of the most common chronic neurological diseases, epilepsy affects more than 50 million people worldwide, and nearly 80% of them live in developing countries (Ngugi, Bottomley, Kleinschmidt, Sander, & Newton, 2010). In Niger, epilepsy is poorly studied, and there is no study realized in the general population determining the incidence and prevalence of this condition. However, a recent study reports a hospital frequency of epilepsy of 29.5%, affecting mainly men and people younger than 20 years (Assadeck et al., 2019). In sub‐Saharan Africa, epilepsy remains a major public health concern with significant economic and socio‐cultural consequences. It is considered as a serious illness because of its alleged contagiousness and incurability resulting in rejection of people with epilepsy by the society responsible for problems of social integration (Maiga et al., 2011; Millogo & Siranyan, 2004). People with epilepsy suffer more from social discrimination than the disease itself. The negative attitudes and beliefs about epilepsy are rife not only in the general population but also in the school environment. In general, school teachers do not receive any formal instruction on epilepsy during their training and most cases of epilepsy tend to occur in childhood and adolescence. We know that epilepsy is the most common neurological disease in school‐age (Seidenberg & Berent, 1992), a period during which children begin and complete a critical part of their social and educational development. Negative attitudes and beliefs about epilepsy of the school teachers can lead to the expulsion of the child and even the nonschooling. Besides, some children with epilepsy may be rejected from their peers for fear of being contaminated. A study conducted in Sakoira in northeastern of Niger regarding knowledge and attitudes about epilepsy of primary and secondary school teachers revealed that 42% of teachers considered epilepsy as a contagious disease and 45% thought that it is not a brain disease (Boubacar et al., 2016). To remedy the problem of school and social discrimination in children with epilepsy, school teachers could play an important role in this affair by providing them information about epilepsy.

The present study was designed firstly to evaluate knowledge, attitudes, and practices about epilepsy of primary and secondary schools teachers in the city of Niamey and secondly to formulate targeted sensitization messages for these teachers.

## 2 METHODS

We conducted a descriptive cross‐sectional survey between May and June 2019 regarding knowledge, attitudes, and practices about epilepsy of primary and secondary school teachers in the city of Niamey, capital of Niger. We have personally visited primary and secondary schools and distributed to school teachers (after obtaining their informed consent and those wishing to participate in the survey) questionnaires (Appendix 1) including questions regarding knowledge, attitudes, and practices about epilepsy that they fill themselves. We ask each participant to complete the questionnaire alone based on their knowledge about epilepsy without resorting to a colleague to limit the influence of each other in the responses. We stay beside the participant when he completes the questionnaire, and at the end, we recover the completed form. At the same opportunity, we take advantage to address awareness messages to the participants regardless of their answers.

The study was approved by the Institutional Review Board of the Faculty of Medicine of Abdou Moumouni University of Niamey (Niger) in accordance with the Declaration of Helsinki.

The data analysis was performed with SPSS software version 20.0 (SPSS Inc.). The qualitative variables were expressed as percentages and the quantitative variables as mean ± standard deviation. The chi‐square test of Pearson was used to compare the proportions of the qualitative variables. *p*‐values < .05 were considered statistically significant.

## 3 RESULTS

### 3.1 Sociodemographic characteristics of the participants

Table 1 details the sociodemographic characteristics of the 145 teachers who completed the questionnaire. The mean age of the participants was 39.57 ± 8.304 (range: 27–69 years) with a predominance of the age group of 30–39 years (46.9%). Women were more represented with a sex ratio of 1.8. Primary school teachers accounted for 37.9% of the sample, and college and lyceum teachers accounted for 27.6% and 34.5%, respectively. One hundred thirty‐six participants (93.8%) were Muslims, while Christians accounted for 6.2%. Hausas were the most represented (43.4%) followed by zarmas (31%).

**Table 1:** Sociodemographic characteristics of the participants

Variables	Number (%)
Sex	
Males	52 (35.9)
Females	93 (64.1)
Sex ratio (Females/males)	1.8
Age (years)	
Mean	39.57 ± 8.304
Range	27 and 69
Mean/males	38.71 ± 9.291
Mean/females	40.05 ± 7.709
<30	14 (9.7)
30–39	68 (46.9)
40–49	43 (29.7)
≥50	20 (13.8)
Religion	
Islam	136 (93.8)
Christianity	9 (6.2)
Ethnie	
Hausa	63 (43.4)
Zarma	45 (31)
Songhaï	13 (9)
Peulh	6 (4.1)
Touareg	3 (2.1)
Kanouri	2 (1.4)
Dandi	2 (1.4)
Arab	1 (0.7)
Goun	2 (1.4)
Ifè	1 (0.7)
Lamba	2 (1.4)
Losso	3 (2.1)
Mina	1 (0.7)
Kabiyè	1 (0.7)
Region of origin	
Niamey	102 (70.3)
Dosso	10 (6.9)
Tillabéry	3 (2.1)
Tahoua	5 (3.4)
Maradi	8 (5.5)
Zinder	5 (3.4)
Agadez	1 (0.7)
Togo	8 (5.5)
Benin	2 (2.1)
Tombouctou	1 (0.7)
Level of teaching	
Primary school	55 (37.9)
College	40 (27.6)
Lyceum (high school)	50 (34.5)

### 3.2 Knowledge about epilepsy of the participants

#### 3.2.1 Have you ever heard about epilepsy?

All participants had previously ever heard about epilepsy, among whom 19 (13.1%) had already attended at least an epileptic seizure. Twenty‐two (15.2%) had heard about epilepsy from a parent or relative of an epileptic person. Thirty‐eight participants (26.2%) had heard about epilepsy of their colleagues or friends, sixteen (11%) of their elders (parents, grandparents), sixteen (11%) through the media, and five (3.4%) through health workers. Twenty‐nine participants (20%) did not specify their information source.

#### 3.2.2 What does epilepsy mean in your language?

Epilepsy means in Hausa: “Falfadiya” or “Borin jaki” or “Mabougui” or “Shigan sandaji.” These terms vary according to the regions of Niger and signify: “sickness who makes fall with the loss of consciousness” or “insanity of the donkey.” Epilepsy means in Zarma: “Tchouro‐tchouro” or “Karga‐fourou” signifying “insanity of the guineafowl” or “agony crisis of the bird.”

#### 3.2.3 How is manifested epilepsy?

One hundred and seven participants (73.8%) had given at least one sign suggestive of an epileptic seizure. The most frequent answers were as follows: “the person falls and makes tremors crisis”; “the person falls and only gets up after urinating”; “the person falls and becomes stiff”; “violent convulsions accompanied by coma”; “convulsions”; “the saliva comes out of the mouth of the patient in quantity, and it throws a little everywhere on the ground”; “tremors and shaking with loss of the consciousness”; “loss of the consciousness and convulsions”; “crisis of the movements of the members of the whole body”; “loss of the consciousness, rejection of gas and urine with the bite of the tongue”; “the person falls and faints”; “loss of the consciousness.”

Eighteen participants (12.4%) had reported signs that did not evoke an epileptic seizure. The most frequent answers were as follows: “high fever”; “fever and headaches”; “strong agitation”; “agitation and mental disorders”; “loss of balance and fall”; “faintness.”

Twenty participants (13.8%) did not answer the question.

#### 3.2.4 What is epilepsy? Is it a contagious disease? Is it a treatable disease?

Table 2 summarizes the participants' responses regarding knowledge about epilepsy. Sixty‐one participants (42.1%) considered epilepsy as a brain disease. However, fourteen participants (9.7%) considered epilepsy to be a witchcraft, five (3.4%) as possession by geniuses, sixteen (11%) as a hereditary disease, and fifty‐six (38.6%) as an impurity. Sixty‐seven participants (46.2%) considered epilepsy to be a contagious disease. The most cited modes of contagion were as follows: “contact with the places of the crisis,” “physical contact with an epileptic person,” and “contact with the saliva of an epileptic person.” One hundred and fifteen participants (79.3%) think that epilepsy is a treatable disease. Forty‐six (31.7%) think that epilepsy is treatable by traditional medicine and or maraboutage while 38 (26.2%) think of treatment by modern medicine. Thirty‐one (21.4%) think that epilepsy is treatable by both modern medicine and traditional medicine and or maraboutage.

**Table 2:** Knowledge about epilepsy of the participants

Variables	Number (%)
Is epilepsy	
Psychiatric illness?	23 (15.9)
Insanity?	3 (2.1)
Impurity?	56 (38.6)
Brain disease?	61 (42.1)
Hereditary disease?	16 (11)
Witchcraft?	14 (9.7)
Possession by geniuses?	5 (3.4)
Epilepsy is a contagious disease?	
Yes	67 (46.2)
No	67 (46.2)
Do not know	1 (0.7)
Contagion mode	
Contact with the saliva of an epileptic?	10 (6.9)
Contact with the blood of an epileptic?	3 (2.1)
Contact with places of crisis?	38 (26.2)
Breathing the gas emitted during the crisis?	4 (2.8)
Physical contact with an epileptic?	24 (16.6)
By eating foods touched by an epileptic?	4 (2.8)
Epileptic parents?	4 (2.8)
Is epilepsy a treatable disease?	
Yes	115 (79.3)
No	10 (6.9%)
Did not answer	20 (13.8%)
By modern medicine	38 (26.2)
By traditional medicine and or maraboutage	46 (31.7)
By combination of traditional and modern medicine	31 (21.4)

Epilepsy is essentially considered to be a brain disease by teachers aged 30–39 years (*p* = .024) and women (*p* < .001) (Table 3). Men think that epilepsy is an impurity (*p* = .006). Teachers aged 40–49 years believe that epilepsy is transmitted by breathing the gas emitted during the seizure (*p* = .014) and men think that transmission is done by eating foods touched by an epileptic person (*p* = .008). We found no relationship between teachers' religious beliefs and their knowledge about epilepsy (Table 4). College teachers believe that epilepsy is transmitted by physical contact with an epileptic person (*p* = .003), while primary school teachers believe that epilepsy is transmitted by contact with the places of the crisis (*p* < .001). Primary school teachers considered epilepsy as a brain disease (*p* < .001) while college teachers thought of witchcraft (*p* = .003) and lyceum teachers an impurity (*p* < .001).

**Table 3:** Knowledge about epilepsy of the participants by sex and age group

Variables	Sex			Age group				
	Males (*n* = 52)	Females (*n* = 93)	*p*	<30 (*n* = 14)	30–39 (*n* = 68)	40–49 (*n* = 43)	≥50 (*n* = 20)	*p*
Psychiatric illness	11 (21.2%)	12 (12.9%)	.192	3 (21.4%)	12 (17.6%)	5 (11.6%)	3 (15%)	.783
Insanity	3 (5.8%)	0	.020	0	1 (1.5%)	0	2 (10%)	.051
Impurity	28 (53.8%)	28 (30.1%)	.006	7 (50%)	19 (27.9%)	20 (46.5%)	10 (50%)	.054
Brain disease	13 (25%)	48 (51.6%)	<.001	2 (14.3%)	37 (54.4%)	16 (37.2%)	6 (30%)	.024
Hereditary disease	10 (19.2%)	6 (6.5%)	.020	2 (14.3%)	9 (13.2%)	3 (7%)	2 (10%)	.810
Witchcraft	7 (13.5%)	7 (7.5%)	.240	0	7 (10.3%)	2 (4.7%)	5 (25%)	.042
Possession by geniuses	3 (5.8%)	2 (2.2%)	.259	2 (14.3%)	0	2 (4.7%)	1 (5%)	.054
Contagious disease	18 (34.6%)	49 (52.7%)	.073	8 (57.1%)	33 (48.5%)	21 (48.8%)	5 (25%)	.415
Contact with the saliva of an epileptic	4 (7.7%)	6 (6.5%)	.804	1 (7.1%)	5 (7.4%)	4 (9.3%)	0	.594
Contact with the blood of an epileptic	1 (1.9%)	2 (2.2%))	.904	0	2 (2.9%)	1 (2.3%)	0	.811
Contact with places of crisis	9 (17.3%)	29 (31.2%)	.049	3 (21.4%)	23 (33.8%)	10 (23.3%)	2 (10%)	.166
Breathing the gas emitted during the crisis	2 (3.8%)	2 (2.2%)	.573	2 (14.3%)	0	2 (4.7%)	0	.014
Physical contact with an epileptic	10 (19.2%)	14 (15.1%)	.572	1 (7.1%)	11 (16.2%)	10 (23.3%)	2 (10%)	.418
By eating foods touched by an epileptic	4 (7.7%)	0	.008	0	2 (2.9%)	2 (4.7%)	0	.678
Epileptic parents	1 (1.9%)	3 (3.2%)	.624	0	2 (1.9%)	1 (2.3%)	1 (5%)	.850

**Table 4:** Knowledge about epilepsy of the participants by religion and level of teaching

Variables	Religion			Level of teaching			
	Islam (*n* = 136)	Christianity (*n* = 9)	*p*	Primary school (*n* = 55)	College (*n* = 40)	Lyceum (*n* = 50)	*p*
Psychiatric illness	20 (14.7%)	3 (33.3%)	.138	9 (16.4%)	7 (17.5%)	7 (14%)	.896
Insanity	3 (2.2%)	0	.648	0	1 (2.5%)	2 (4%)	.338
Impurity	54 (39.7%)	2 (22.2%)	.268	0	24 (60%)	32 (64%)	<.001
Brain disease	58 (42.6%)	3 (33.3%)	.534	42 (76.4%)	9 (22.5%)	10 (20%)	<.001
Hereditary disease	14 (10.3%)	2 (22.2%)	.288	5 (9.1%)	5 (12.5%)	6 (12%)	.826
Witchcraft	14 (10.3%)	0	.301	1 (1.8%)	9 (22.5%)	4 (8%)	.003
Possession by geniuses	4 (2.9%)	1 (11.1%)	.205	2 (3.6%)	1 (2.5%)	2 (4%)	.919
Contagious disease	62 (45.6%)	5 (55.6%)	.909	30 (54.5%)	20 (50%)	17 (34%)	.220
Contact with the saliva of an epileptic	8 (5.9%)	2 (22.2%)	.085	1 (1.8%)	4 (10%)	5 (10%)	.116
Contact with the blood of an epileptic	3 (2.2%)	0	.634	1 (1.8%)	2 (5%)	0	.250
Contact with places of crisis	34 (25%)	4 (44.4%)	.290	26 (47.3%)	6 (15%)	6 (12%)	<.001
Breathing the gas emitted during the crisis	4 (2.9%)	0	.581	1 (1.8%)	1 (2.5%)	2 (4%)	.761
Physical contact with an epileptic	23 (16.9%)	1 (11.1%)	.562	2 (3.6%)	11 (27.5%)	11 (22%)	.003
By eating foods touched by an epileptic	3 (2.2%))	1 (11.1%)	.145	0	2 (5%)	2 (4%)	.259
Epileptic parents	3 (2.2%)	1 (11.1%)	.145	2 (3.6%)	2 (5%)	0	.320

### 3.3 Participants' attitudes about epilepsy

#### 3.3.1 What do you do when someone has an epileptic seizure?

The most frequent answers are presented.

“I'm afraid I prefer to get away from the patient (14.5%).” “Pour him the water on the head (3.4%).” “Nothing, I observe (11%).” “Burn the places after the crisis (2.1%).” “Maintain the person on the side until the crisis passes (14.5%).” “Avoid to approach him and not touch the places of the crisis (2.1%).” “Withdrawal dangerous objects around the person (17.9%).” “Call for help (3.4%).” “Transport the person to the hospital (3.4%).”

#### 3.3.2 What do you avoid to do?

The most frequent answers are presented.

“Physical contact with the patient, contact with the places of the crisis (28.3%).” “Contact with the patient's saliva (2.7%).” “Abandon the patient (4.8%).” “Put something in the patient's mouth (4.8%).” “Make noises around the patient (6.2%).” “Leave dangerous objects around the patient (7.6%).”

### 3.4 Participants' suggestions

The most frequent answers are presented.

“Do not let epileptic persons drive, walk around water and fire (2.1%).” “Make a treatment by modern medicine (6.9%).” “Morally support people with epilepsy and avoid stigma (4.1%).” “Do not let the person walk long alone in the city (2.1%).”

## 4 DISCUSSION

In this study, we evaluate knowledge, attitudes, and practices about epilepsy among primary and secondary school teachers in the city of Niamey, capital of Niger. Because epilepsy mainly affects people younger than 20 years in Niger (Assadeck et al., 2019), we have chosen to specifically conduct the survey among primary and secondary school teachers who are responsible for the social and educational development of people in this age group and to transmit them targeted awareness messages about epilepsy so that they can transmit these messages to a larger number of students to avoid the rejection of epileptic students by their peers. The majority of teachers (62.1%) who completed the questionnaire had a bachelor degree in education signifying a high level of education. However, the study results revealed a low level of general knowledge about epilepsy among respondents, of whom only 42.1% think that epilepsy is a brain disease. Epilepsy is considered to be a psychiatric illness by 15.9% of respondents, an impurity by 38.6% of teachers, and a contagious disease by 46.2% of respondents. In the study conducted in Sakoira in northeastern of Niger (Boubacar et al., 2016), the percentage of the primary and secondary school teachers of this city considering epilepsy as a brain disease seems to be higher than that of teachers in the city of Niamey (52% vs. 42.1%; *p* > .2). In the same study, 42% of respondents considered epilepsy to be a contagious disease, whereas in the present study 46.2% of teachers think that epilepsy is contagious (*p* > .2). In other African studies conducted among school teachers, epilepsy is considered as a contagious disease in 28.2–39% of teachers surveyed and in 22.8–53.4% considered as a brain disease (Ategbo et al., 2014; Birbeck, Chomba, Atadzhanov, Mbewe, & Haworth, 2006; Maiga et al., 2015). The main routes of transmission reported in these studies were saliva, sweat, and urine. In this study, the main transmission routes reported were the contact with the places of crisis, physical contact with an epileptic person, and contact with saliva. On the other hand, low proportions ranging from 1% to 11.9% of teachers considering epilepsy as a contagious disease have been reported in other African studies (Gebrewold, Enquselassie, Teklehaimanot, & Gugssa, 2016; Millogo & Siranyan, 2004; Shehata & Mahran, 2010). In some Asian studies, epilepsy is considered to be contagious in 0.2–15.3% of teachers surveyed and in 44.4–83.6% as a brain disease (Alkhamra, Tannous, Hadidi, & Alkhateeb, 2012; Lee, Lee, Chung, Yun, & Choi‐Kwon, 2010; Thacker, Verma, Ji, Thacker, & Mishra, 2008). Epilepsy is considered to be a psychiatric illness by 15.9% of teachers surveyed in our study. This percentage is higher than the percentages observed among South Korean teachers (3.5%) and Egyptian teachers (10.2%) and lower than that observed among Ethiopian teachers (25.9%) (Gebrewold et al., 2016; Lee et al., 2010; Shehata & Mahran, 2010). These differences could be explained by several parameters such as the socio‐cultural context, the religious beliefs of the population, and the education system. In Niger, several infectious and tropical diseases and deficiency diseases are taught in secondary schools, but epilepsy is not taught although it is common. This would explain the low level of knowledge about epilepsy among school teachers in Niger.

Although all respondents in our study had ever heard about epilepsy, 12.4% of the respondents were unable to give a major clinical sign of an epileptic seizure. Epilepsy is considered to be an incurable disease by 6.9% of respondents in our study. This proportion is lower than those reported in Burkina Faso (13.5%), in Mali (34.8%), in Jordan (52%), in India (21.4%), in South Korea (22%), and to that reported in a previous study from Niger (35%) (Alkhamra et al., 2012; Boubacar et al., 2016; Lee et al., 2010; Maiga et al., 2015; Millogo & Siranyan, 2004; Thacker et al., 2008). Of the respondents who consider epilepsy as a curable disease, 21.4% of them think that epilepsy can be treated by both modern and traditional medicine. On the other hand, 31.7% think that epilepsy is treated only by traditional medicine, while 26.2% only advise modern medicine. In Burkina Faso, only 15% of teachers think that epilepsy is treated only by modern medicine, and 56% by the combination of modern and traditional medicine (Millogo & Siranyan, 2004). In Mali, 83.7% of teachers advise modern medicine (Maiga et al., 2015).

Although the general level of knowledge about epilepsy was low among the respondents in our study, 32.4% of teachers reported appropriate attitudes when someone has an epileptic seizure such as “Maintain the person on the side until the crisis passes,” “Withdrawal dangerous objects around the person.” This percentage of appropriate attitudes found in our study is comparable to that found in Malian teachers (35%) and lower than that found in teachers in Burkina Faso (40%) (Maiga et al., 2015; Millogo & Siranyan, 2004).

At the end of the survey, school teachers' responses demonstrate that they were less knowledgeable about epilepsy. Many school teachers emphasized the impact of negative religious and cultural beliefs about epilepsy in their attitude toward people with epilepsy. Besides, many school teachers had shown their satisfaction with the information provided concerning epilepsy, and some of them would like to have more information on this superstitious and mythical disease. Therefore, this underlines the need to integrate well‐targeted information modules on epilepsy as well as the adapted gestures to perform when a child has an epileptic seizure. School teachers underlined the interest of stopping to send at home the students with epilepsy and helping them in the school learning and social integration by changing the negative attitudes of students without medical conditions and promoting positive attitudes toward students with epilepsy. Considering that epilepsy is a common disease in school‐age, it is very important to minimize the negative consequences of this disease in a school environment because it can lead to problems of schooling for children with epilepsy.

## 5 CONCLUSION AND RECOMMENDATIONS

In this study assessing knowledge, attitudes, and practices about epilepsy, we find a low overall level of knowledge about this condition among school teachers in the city of Niamey. The study results encourage special attention in the epileptic children in school‐age to avoid the rejection of these children by the society responsible for problems of social integration. This low level of general knowledge about epilepsy among school teachers, knowing that epilepsy is common among school‐age children, justifies the need to integrate into the training modules of the future school teachers information modules related to epilepsy to enable them to acquire know‐how about epilepsy. It is also necessary to organize continuing education seminars for teachers already in post. The study recommends further research to evaluate knowledge, practices, and attitudes regarding epilepsy among school teachers of other cities of Niger and at the same opportunity to address targeted awareness messages for these teachers. The study also recommends conducting research on knowledge, practices, and attitudes of health workers. The study also recommends public education about epilepsy using possible avenues such as the integration of information modules regarding epilepsy into the national education program, the mass media especially radio and television which are an important source of information in most developing countries, and awareness campaigns to change any misconceptions about epilepsy and to promote positive attitudes toward people with epilepsy.

## CONFLICT OF INTEREST

All authors have read and agreed to the content of the manuscript. None of the authors has any conflict of interest to declare. The authors declared no potential conflicts of interest with respect to the research, authorship, and/or publication of this article. We confirm that this manuscript has not previously been published and is not being considered for publication by another journal. The authors alone are responsible for the content and writing of the paper.

## Data Availability

All data generated or analyzed during this study are included in this published article.
